# Cellular specificity of mitochondrial and immunometabolic features in major depression

**DOI:** 10.1038/s41380-022-01473-2

**Published:** 2022-02-18

**Authors:** Jelena Brasanac, Stefanie Gamradt, Christian Otte, Yuri Milaneschi, Anna S. Monzel, Martin Picard, Stefan M. Gold

**Affiliations:** 1 Charité—Universitätsmedizin Berlin, Klinik für Psychiatrie und Psychotherapie, Campus Benjamin Franklin, Berlin, Germany; 2 Department of Psychiatry, Amsterdam Public Health and Amsterdam Neuroscience, Amsterdam UMC/Vrije Universiteit, Amsterdam, The Netherlands; 3 Division of Behavioral Medicine, Department of Psychiatry and Neurology, Columbia University, New York, NY USA; 4 Charité—Universitätsmedizin Berlin, Medizinische Klinik m.S. Psychosomatik, Campus Benjamin Franklin, Berlin, Germany; 5 Institut für Neuroimmunologie und Multiple Sklerose (INIMS), Zentrum für Molekulare Neurobiologie, Universitätsklinikum Hamburg-Eppendorf, Hamburg, Germany

**Keywords:** Biological techniques, Molecular biology

## To the Editor:

Converging lines of evidence from human studies and experimental models have recently suggested a putative pathogenetic role of mitochondrial biology in neuropsychiatric disorders including depression. However, mitochondrial regulation is tissue-specific and varies substantially between subsets of cell populations, particularly in the immune system, where mitochondrial bioenergetics and dynamics drive cell function. Thus, while the peripheral immune system is an attractive biomarker candidate due to its easy accessibility in humans and its likely involvement in mood disorders, investigations in this area will require detailed workup of cellular specificity and functional implications to gain insight into potentially druggable pathobiological substrates.

In this journal, Scaini et al. [1] reported altered expression of key mitochondrial proteins including Mfn-2, short Opa-1 and Fis-1 in peripheral blood mononuclear cells (PBMCs) from a cross-sectional cohort of MDD patients compared to healthy controls. Moreover, their analyses suggested that expression of some of these mitochondrial markers might be linked to low grade inflammation (as determined by CRP levels). While mitochondrial functions were not tested directly in this study, the differential expression of these proteins in PBMCs supports earlier reports of altered cellular respiration of PBMCs obtained from patients with MDD (e.g. [2]). Moreover, the study adds to converging evidence from a recent metabolomics study [3] and a genetic investigation using Mendelian randomization [4], which implicated mitochondrial biology in the pathogenesis of depression.

**Fig. 1:**
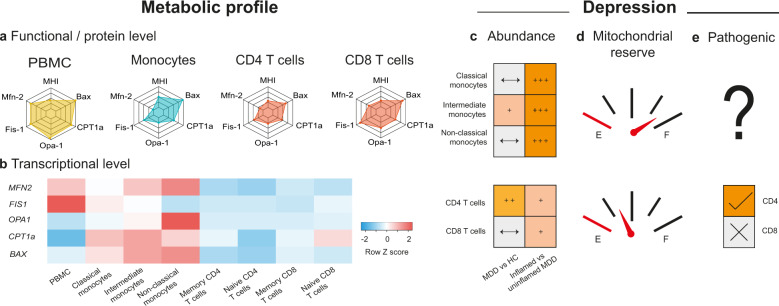
Cellular specificity of mitochondrial phenotype in cell subsets of the peripheral immune system in major depression. **a** On a functional and protein level, mitochondrial bioenergetics and dynamics differ substantially between bulk peripheral blood mononuclear cells (PBMCs) in comparison to purified subsets of immune cells such as monocytes, CD4 T cells, or CD8 T cells. Mitochondrial health index (MHI) taken from [6], abundance of key mitochondrial proteins Mfn-2, Fis-1, Opa-1, CPT1a and Bax based on data obtained from https://www.genecards.org/. **b** Further complexity is added on a transcriptional level when exploring subsets of monocyte and T cell population. Heatmap shows cell-specific gene expression from RNA-seq data based on molecularly defined, flow-sorted immune cell subpopulations in the HumanProteinAtlas [7]. Red color indicates higher expression, blue color lower expression (normalized within row). **c** Based on immunophenotyping data comparing patients with major depressive disorder (MDD) and healthy controls (HCs), subsets of monocytes and T cells are more frequent in the peripheral immune system in depression. This effect is particularly pronounced in a subgroup of patients, as shown by comparisons between “uninflamed” and “inflamed” subtypes of MDD (based on clustering of peripheral cellular immune markers), see [8]. **d** In MDD, impairments of mitochondrial reserve (as measured by oxygen consumption rate) were more pronounced in purified T cells than in monocytes (based on [9]). **e** Mitochondrial dysfunction in CD4 T cells specifically has been demonstrated to induce depression/anxiety-like behavior in mice (based on [10]).

However, it is becoming increasingly clear that different cell types harbor unique mitochondrial phenotypes and functions. In the immune system, mitochondrial bioenergetics and dynamics are key drivers of immune cell differentiation and function [5] and—consequently—there are very large differences in the mitochondrial phenotypes among different cell lineages (e.g., monocytes, B and T lymphocytes) and activation or differentiation states (e.g., memory vs naïve) [6]. Mirroring differences on a functional and protein level (Fig. 1a), RNA expression levels of key mitochondrial genes reported to differ between MDD and controls also vary by up to 37-242% among immune cell subtypes, and—importantly—between these subtypes vs PBMCs (Fig. 1b) [7].

This point is relevant to depression, because numerous studies have shown that MDD is associated with shifts in immune subset composition (see Fig. 1c). As an example, the largest and most recent immunophenotyping study in MDD [8] showed a number of enumerative differences including higher circulating numbers of monocyte subsets and CD4^+^ T cells in MDD compared to controls. This immune signature was also linked to elevated serum markers such as CRP, characterizing a subgroup of “inflamed” depression. Moreover, the degree of alterations in mitochondria such as respiratory chain function appears to differ between cell populations of the adaptive and innate immune system when compared between patients with MDD and closely matched healthy controls [9] (see Fig. 1d). Here, group differences in mitochondrial respiration were much more pronounced in T cells compared to monocytes. Importantly, such lineage-specific differences within the immune system might be biologically relevant in depression as an elegant experimental study in mice [10] demonstrated that adoptive transfer of CD4^+^ T cells (but not CD8^+^ T cells) from stressed mice was sufficient to induce depression/anxiety-like behavior in (unstressed) recipient mice (see Fig. 1e). In a series of well-controlled experiments, the authors established that a defect in mitochondrial fusion specifically in CD4^+^ T cells was the key mechanism for this effect.

Thus, when examining cellular or molecular markers in bulk-PBMCs, shifts in the composition of the immune cell subsets within the PBMC sample could mask, blunt, or maybe even drive apparent group differences in case-control studies of MDD. Future studies should therefore consider the inherent cellular specificity and functional implications of immune cell mitochondria, which will help to separate what constitutes an epiphenomenon from robust bioenergetic features and potential therapeutic targets of MDD.
